# Association of serum glial fibrillary acidic protein with progression independent of relapse activity in multiple sclerosis

**DOI:** 10.1007/s00415-024-12389-y

**Published:** 2024-04-26

**Authors:** Igal Rosenstein, Anna Nordin, Hemin Sabir, Clas Malmeström, Kaj Blennow, Markus Axelsson, Lenka Novakova

**Affiliations:** 1 Department of Clinical Neuroscience, Institute of Neuroscience and Physiology at Sahlgrenska Academy, University of Gothenburg, Blå Stråket 7, 413 45 Gothenburg, Sweden; 2 Department of Neurology, Region Västra Götaland, Sahlgrenska University Hospital, Mölndal, Sweden; 3 Clinical Neurochemistry Laboratory, Sahlgrenska University Hospital, Mölndal, Sweden; 4 Department of Psychiatry and Neurochemistry, Institute of Neuroscience and Physiology, University of Gothenburg, Mölndal, Sweden; 5 Paris Brain Institute, ICM, Pitié-Salpêtrière Hospital, Sorbonne University, Paris, France; 6 Neurodegenerative Disorder Research Center, Division of Life Sciences and Medicine, and Department of Neurology, Institute On Aging and Brain Disorders, University of Science and Technology of China and First Affiliated Hospital of USTC, Hefei, People’s Republic of China

**Keywords:** Biomarkers, Disability worsening, Glial fibrillary acidic protein, Neurofilament light, Synthetic MRI

## Abstract

**Objective:**

Insidious disability worsening is a common feature in relapsing–remitting multiple sclerosis (RRMS). Many patients experience progression independent of relapse activity (PIRA) despite being treated with high efficacy disease-modifying therapies. We prospectively investigated associations of body-fluid and imaging biomarkers with PIRA.

**Methods:**

Patients with early RRMS (*n* = 104) were prospectively included and followed up for 60 months. All patients were newly diagnosed and previously untreated. PIRA was defined using a composite score including the expanded disability status scale, 9-hole peg test, timed 25 foot walk test, and the symbol digit modalities test. Eleven body fluid and imaging biomarkers were determined at baseline and levels of serum neurofilament light (sNfL) and glial fibrillary acidic protein (sGFAP) were also measured annually thereafter. Association of baseline biomarkers with PIRA was investigated in multivariable logistic regression models adjusting for clinical and demographic confounding factors. Longitudinal serum biomarker dynamics were investigated in mixed effects models.

**Results:**

Only sGFAP was significantly higher in PIRA at baseline (median [IQR] 73.9 [60.9–110.1] vs. 60.3 [45.2–79.9], *p* = 0.01). A cut-off of sGFAP > 65 pg/mL resulted in a sensitivity of 68% and specificity of 61%, to detect patients at higher risk of PIRA. In a multivariable logistic regression, sGFAP > 65 pg/mL was associated with higher odds of developing PIRA (odds ratio 4.3, 95% CI 1.44–12.86, *p* = 0.009). Repeated measures of sGFAP levels showed that patients with PIRA during follow-up had higher levels of sGFAP along the whole follow-up compared to stable patients (*p* < 0.001).

**Conclusion:**

Determination of sGFAP at baseline and follow-up may be useful in capturing disability accrual independent of relapse activity in early RRMS.

**Supplementary Information:**

The online version contains supplementary material available at 10.1007/s00415-024-12389-y.

## Background

Multiple sclerosis (MS) is an immune-mediated disease in which both the adaptive and innate immune systems play an important role. The adaptive immune system is often associated with clinical relapses and the formation of focal inflammatory lesions. Conversely, accumulating data have recently provided strong evidence for the involvement of the innate immune system in creating a low-grade inflammatory milieu that is associated with worsening of neurodegeneration [1], a process also known as smouldering MS [2]. Smouldering MS is now recognized as the main biological process behind the clinical phenotype of irreversible disability accrual unrelated to relapses, i.e. progression independent of relapse activity (PIRA) [3–5]. To increase the comparability and interpretation of studies investigating PIRA as an outcome measure, a harmonizing definition has recently been proposed [6]. In addition to a clinically significant elevation in the expanded disability status scale (EDSS), the authors recommended the use of a composite score including significant and sustained changes in the 9-hole peg test (9HPT), timed 25 foot walk test (T25FWT), and the single digit modalities test (SDMT) [6].

The goal of halting relapses and focal lesion formation in MS has been largely achieved with the advent of highly effective disease modifying therapies (DMTs). However, the effect of DMTs on disability worsening is modest [7]. Undoubtedly, the next major challenge will be the development of therapies that influence smouldering MS and its clinical phenotype, i.e. disability worsening that occurs independent of inflammatory disease activity. To that end, recognizing smouldering processes early in the course of the disease and identifying those patients that are at greater risk of developing worsening disability despite having their inflammatory disease activity under control is of high clinical relevance. Therefore, identifying easily accessible biomarkers that can accurately reflect disability worsening and assist in identifying patients with higher risk of developing PIRA is warranted. Particularly, the serum biomarkers neurofilament light (NfL) and glial fibrillary acidic protein (GFAP) have shown some potential in this regard [8, 9].

Cerebrospinal fluid (CSF) GFAP has been long known to reflect disease progression in MS [10–12]. Further, CSF NfL is known to associate mostly with inflammatory disease activity [10, 13], but several studies have also demonstrated an association between CSF NfL and disability worsening [14, 15]. The development of ultrasensitive immunoassays enabled measuring these biomarkers in serum and plasma, rendering GFAP and NfL as potential biomarkers for clinical practice. Serum GFAP (sGFAP) and NfL (sNfL) have been shown to associate with MS disease severity [9, 16–20], although several studies have failed to demonstrate an association between longitudinal measurements and disability worsening over time [21–23].

Our aim was thus to explore the association of various body fluid- and imaging biomarkers with PIRA in a well-defined prospective cohort of patients with early relapsing–remitting MS (RRMS).

## Materials and methods

### Study design

Patients with suspected MS onset were prospectively included in a cohort study at the Multiple Sclerosis Center of Sahlgrenska University Hospital, Gothenburg, Sweden between April 2014 and June 2016. The cohort was previously described [24] and prospectively followed to evaluate biomarkers of neurodegeneration. Inclusion criteria were diagnosis of RRMS according to McDonald criteria [25] and follow-up for at least 3 years. Exclusion criteria were other concomitant neurological, ophthalmological and inflammatory diseases. All patients were newly diagnosed, untreated at the time of inclusion, and started treatment with DMT according to physicians’ recommendation. The dates of the first symptom manifestation, diagnosis, relapses and concomitant disease were recorded at the baseline visit and revised at the end of the study. EDSS, 9-HPT, T25FWT, SDMT, magnetic resonance imaging (MRI) and optical coherence tomography (OCT) were performed at baseline, month 6, 12, 24, 36, 48 and 60. The sampling of CSF was done at baseline whereas serum sampling was performed at baseline and month 12, 24, 36, 48 and 60.

### Evaluation of disability accrual as study endpoints

We investigated two endpoints, sustained confirmed disability worsening (CDW), and PIRA as determined by a composite score, designated PIRA^+^. Sustained CDW was defined as sustained increase in EDSS score by ≥ 1.5, ≥ 1 and ≥ 0.5 if baseline EDSS was 0, 1.0–5.0 and ≥ 5.5, respectively. PIRA^+^ was defined using the following combined outcome parameters: sustained CDW; increase of T25FWT and 9-HPT by > 20%; and increase of SDMT score by > 10% [6]. A PIRA^+^ event was determined only in patients after a confirmation visit > 3 months after the event score, and absence of relapses between baseline and confirmation score. The PIRA event was sustained if it was confirmed at the last study visit. We used a roving baseline EDSS [3] and combined EDSS/T25FWT/9HPT/SDMT approach to identify PIRA with high specificity [6]. PIRMA^+^ was defined similarly to PIRA^+^, with the addition of the absence of signs of inflammatory MRI disease activity (new/enlarging T2 lesions and/or CELs) within three months apart from a PIRA^+^ event.

### Imaging biomarkers

Brain MRI was performed on 3.0 Tesla MRI scanner (Philips Achieva dStream, head coil type with 16 coil channels). All sequences were obtained after a standard dose of iv gadolinium. We acquired conventional T1-weighted, T2-weighted and fluid-attenuated inversion recovery and Synthetic MRI (SyMRI) sequences [26, 27]. A trained neuroradiologist assessed the T2 lesion and contrast-enhancing lesion (CEL) count. The brain parenchymal fraction (BPF) and myelin content (MY) of the brain were created from R1, R2 and PD maps via SyMRI software (version 11.2; SyntheticMR, Linköping, Sweden). These measures enable tracking global brain atrophy and global cerebral demyelination and are CE marked for clinical use in Europe.

OCT with 3D Disc (peripapillary ring scan) and 3D Wide (scan of whole retina) scans were performed by a trained nurse on a Topcon 3D OCT-2000 (Topcon Medical Systems, Tokyo prefecture, Japan). The peripapillary retinal nerve fiber layer (pRNFL) and macular ganglion cell inner plexiform layer (mGCIPL) thickness were obtained using the software IMAGEnet6. The OCT scans were quality-controlled according to the validated OSCAR-IB criteria [28] by a trained neuro-ophthalmologist and excluded if they were of unsatisfactory quality. We used average measures of pRNFL and mGCIPL of both eyes for each patient.

### Body fluid biomarkers

All biomarker analyses were performed by certified laboratory technicians in the Clinical Neurochemistry Laboratory at the Sahlgrenska University Hospital. The samples were handled according to the consensus protocol of the BioMS-EU network for CSF biomarker research in MS [29]. Blood and CSF samples were collected simultaneously, processed onsite to isolate serum, aliquoted, and frozen at −80 °C. All analyses were performed at room temperature. The analysis including oligoclonal bands (OCB), IgG index, CSF NfL, CSF GFAP and CSF total tau (T-tau) were performed directly after sampling, as described previously [24]. Briefly, the detection of OCB was performed with isoelectric focusing (IEF). CSF NfL was measured with a sensitive sandwich ELISA method (NF-light1ELISA kit, UmanDiagnostics AB, Umeå, Sweden), the lower limit of quantification (LLoQ) was 31 ng/L. CSF GFAP was measured with an in-house ELISA [30], the LLoQ was 16 pg/mL. CSF T-tau was measured with an ELISA (INNOTEST hTAU Ag, Fujirebio, Ghent, Belgium), the LLoQ was 75 ng/L. The concentrations of serum GFAP and NfL, performed after defrosting, were measured with the Single Molecule Array (Simoa®) NEUROLOGY 2-PLEX B Kit, Product number: 103520, from Quanterix (Billerica, MA, USA) [31]. The LLoQ for serum GFAP and NfL was 29.4 and 1.41 pg/mL, respectively. Serum samples of GFAP and NfL below the LLoQ level were designated the value of fLLoQ. The intra- and interassay coefficients of variation of all analyses were below 10%.

### Statistical analysis

Data are presented as mean ± SD or as median and interquartile range (IQR), as appropriate. Data distribution was assessed with the Shapiro-Wilks test. The Mann–Whitney *U* test, unpaired *T* test, χ^2^ test, and Fisher’s exact test were used for group comparisons, as appropriate. Biomarker correlations were calculated using the spearman correlation coefficient. Biomarker correlations with age were determined with the spearman correlation coefficient and the association with sex was determined with the Mann–Whitney *U* test. For biomarkers that are known to be age-dependent (NfL, GFAP, and Tau), analysis of covariance (ANCOVA) was performed to adjust for age. The Mann–Whitney *U* test was also used for all comparisons of CSF and serum biomarkers stratified by CDW and PIRA^+^ status.

Based on the results of CSF and serum biomarker comparisons, we selected sGFAP for further analysis. To determine the most appropriate cut-off value for sGFAP to predict CDW and PIRA^+^, we performed a receiver operating characteristic (ROC) curve analysis, and calculated the area under the curve (AUC), using the Wilson/Brown method. We calculated the most discriminating cut-off value according to the Youden index, and thereafter computed the sensitivity and specificity to detect CDW and PIRA^+^. Next, we investigated the associations of the calculated cut-off values with CDW and PIRA^+^ in univariable and multivariable logistic regression models. The adjusted odds ratios (aOR) along with corresponding 95% confidence intervals (CI) and *p*-values were calculated. Based on previous investigations on prognostic factors, we adjusted the models for the following potential confounding covariates: age at baseline, sex, disease duration prior to baseline, roving EDSS as defined above, brain MRI characteristics (baseline T2 lesions and CEL),and exposure to high-efficacy (he) DMT. Natalizumab, fingolimod, and alemtuzumab were classified as heDMT, whereas teriflunomide, dimethyl fumarate, or platform therapies, were grouped as low/moderate efficacy DMT. Due to the exploratory nature of the study, no correction for multiple comparisons in the logistic regression were performed.

The same analyses described above were performed for the purpose of sensitivity analyses, investigating progression independent of relapse and MRI activity (PIRMA).

Next, we investigated the longitudinal dynamics of repeated measures of serum biomarkers using mixed-effects analysis (alpha = 0.05). Serum biomarkers were log2-transformed, and estimates and corresponding 95%CIs were back-transformed. We performed both simple and multivariable models, adjusted for age, sex, disease duration, and DMT strategy. The Bartlett´s test of sphericity was used to test the null hypothesis that the residual covariance matrix is proportional to an identity matrix. The reported model *p* values were derived from the fixed effects (type III) analysis. Tukey’s method was used for multiple comparisons to compare biomarker levels at each time point between the groups, and the adjusted *p* value is reported.

Statistical significance was assumed at *p* < 0.05 unless otherwise specified. All statistical analyses and figures were performed/created with IBM SPSS version 28.0.1.0 (Armonk, NY: IBM Corp. 2011) and GraphPad prism version 9.1.0.

### Ethical standards

All patients participated voluntarily in the study and provided written informed consent. The study conformed to the Code of Ethics of the World Medical Association (Declaration of Helsinki). The Regional Ethics Review Board in Gothenburg, Sweden, approved the study (Reference number 895-13).

## Results

### Demographic and clinical characteristics of RRMS patients

The study population was comprised of 104 patients with early RRMS (Table 1), the majority (76%) of whom were female and the median (IQR) age was 34 years (27–39). Thirteen (12.5%) RRMS patients demonstrated sustained CDW, whereas 40 (38.5%) patients exhibited PIRA^+^. Patients with PIRA^+^ were significantly older compared to patients without PIRA^+^ (*p* = 0.014). Overall, 97 patients (93.3%) completed the 60-month follow-up. There were no significant differences between the two groups in terms of sex, disease duration, baseline and roving EDSS, MRI measures, and treatment strategy, but patients with PIRA^+^ had significantly higher EDSS at last visit (*p* < 0.001). Demographical and clinical characteristics of the study population are presented in Table 1.

**Table 1:** Clinical and demographical characteristics of RRMS patients included in the study and dichotomized by non-PIRA+ /PIRA+ status at follow-up

Variable	Total RRMS patients (*n* = 104)	Non-PIRA^+^ (*n *= 64)	PIRA^+^ (*n* = 40)	*p*-value
Age, median (IQR)	34 (27–39)	32 (26–37.75)	35.5 (29.25–46)	**0.014**^**a**^
Sex (Female), *n* (%)	79 (76)	46 (71.9)	33 (82.5)	0.25^b^
Disease duration from symptom onset to inclusion, m, mean ± SD	2.2 ± 4.5	1.8 ± 4.15	2.7 ± 5.1	0.4^c^
Onset EDSS, median (IQR)	2 (1–2.5)	2 (1–2.5)	2 (1–2.5)	0.79^a^
Roving EDSS, median (IQR)	1.5 (0–2)	1 (0–2)	1.5 (0–2)	0.15^a^
EDSS at last visit, median (IQR)	1.5 (0–2)	0.5 (0–2)	2 (1.13–2.5)	** < 0.001**^**a**^
BL 9HPT sec, median (IQR)	19.84 (18.28–22.12)	19.76 (18.42–22.22)	19.84 (18.21–21.51)	0.51^a^
BL T25FWT sec, median (IQR)	3.9 (3.5–4.25)	3.9 (3.55–4.25)	3.9 (3.5–4.35)	0.81^a^
BL SDMT, mean ± SD	56.5 ± 11.7	56.8 ± 13.1	55.9 ± 9.3	0.7^c^
BL T2W lesions, *n* (%)				0.15^b^
< 10	57 (54.8)	39 (60.9)	18 (45)	
≥ 10	47 (45.2)	25 (39.1)	22 (55)	
BL CEL, *n* (%)	52 (50)	33 (51.6)	19 (47.5)	0.84^b^
BL Myelin content (ml), median (IQR)	182.2 (168.5–198.9)	180.8 (166.6–202.1)	182.5 (175.3–198.1)	0.4^a^
BL BPF (%), median (IQR)	0.89 (0.87–0.92)	0.9 (0.87–0.92)	0.88 (0.86–0.92)	0.12^a^
IgG OCBs ≥ 2, *n* (%)	98 (94.2)	59 (92.2)	39 (97.5)	1.0^b^
IgG index, median (IQR)	0.85 (0.64–1.2)	0.83 (0.63–1.18)	0.93 (0.67–1.4)	0.43^a^
CSF NfL ng/L, median (IQR)	640 (400–1760)	580 (365–1890)	655 (575–1753)	0.3^a^
Tau ng/L, median (IQR)	222.5 (168.5–314.3)	216 (174–312.5)	230 (154.5–319)	0.77^a^
CSF GFAP ng/L, median (IQR)	290 (200–390)	260 (195–360)	305 (217.5–437.5)	0.097^a^
sNfL pg/mL, median (IQR)	9.95 (6.6–16.7)	9.05 (6.52–15.35)	11.35 (7.5–18.1)	0.14^a^
sGFAP pg/mL, median (IQR)	65.1 (52.3–88.3)	60.3 (45.2–79.9)	73.9 (60.9–110.1)	**0.01**^**a**^
Mean RNFL μm, median (IQR)	98.5 (94.25–106.1)	99 (95.63–107.8)	98.25 (92–104.5)	0.3^a^
Mean GCIPL μm, median (IQR)	70.7 (66.3–73.7)	71.05 (65.4–73.7)	69.8 (66.3–73.2)	0.45^a^
Exposure to heDMT, *n* (%)	42 (40.4)	24 (37.5)	18 (45)	0.54^b^
DMT, *n* (%)				0.76^b^
Injectables	7 (6.7)	5 (7.8)	2 (5)	
DMF	43 (41.3)	29 (45.3)	14 (35)	
Teriflunomide	5 (4.8)	2 (3.1)	3 (7.5)	
Fingolimod	8 (7.7)	5 (7.8)	3 (7.5)	
Natalizumab	31 (29.8)	18 (28.1)	13 (32.5)	
Alemtuzumab	10 (9.6)	5 (7.8)	5 (12.5)	

RRMS = relapsing–remitting multiple sclerosis; PIRA = progression independent of relapse activity; SD = standard deviation; BL = baseline; EDSS = expanded disability status scale; IQR = interquartile range; MRI = magnetic resonance imaging; T2W = T2-weighted; CEL = contrast-enhancing lesion; BPF = brain parenchymal fraction; IgG = immunoglobulin G; OCB = oligoclonal bands; CSF = cerebrospinal fluid; NfL = neurofilament light; GFAP = glial fibrillary acidic protein; s = serum; RNFL = retinal nerve fiber layer; GCIPL = ganglion-cell inner plexiform layer; he = high-efficacy; DMT = disease modifying therapy; DMF = dimethyl fumarate;

Data are shown as median and interquartile range unless otherwise specified

^a^ Mann–Whitney *U* test

^b^ Fisher’s exact test or Pearson chi-square test

^c^Unpaired *T*-test

Bold text indicates *p* values < 0.05

### Association of baseline body-fluid biomarkers with PIRA

**Fig. 1:**
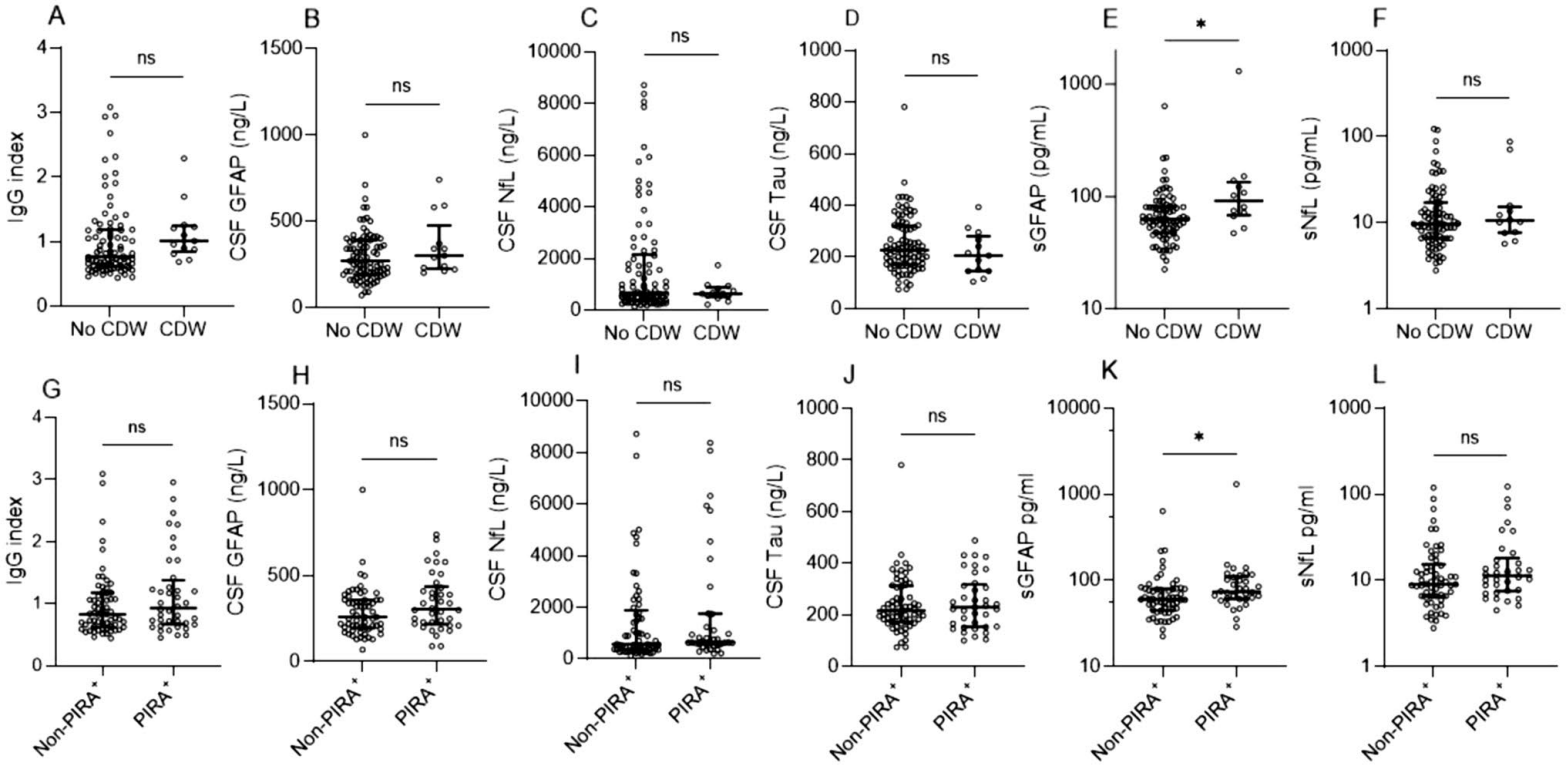
Scatter plots showing CSF and serum biomarker concentrations at baseline in patients who remained stable compared to those who experienced a sustained CDW (**A–F**) and sustained PIRA^+^ event (**G–L**) during follow-up. Biomarker concentrations at baseline were compared with the Mann–Whitney *U* test. Bar represents median and error bars represent interquartile range. CDW = confirmed disability worsening; PIRA = progression independent of relapse activity; CSF = cerebrospinal fluid; Ig = immunoglobulin; NfL = neurofilament light; GFAP = glial fibrillary acidic protein; s = serum. ns = non-significant; * *p* < 0.05

None of the measured biomarkers at baseline significantly correlated with age, and none was associated with sex. Supplemental Figure 1 shows the correlations of all biomarkers. RRMS patients who developed sustained CDW and PIRA^+^ during follow-up had significantly higher sGFAP concentrations at baseline compared to those who remained stable (median [IQR] 91.5 [68.2–134.3] vs 62.75 [47.6–81.7], *p* = 0.013; and 73.9 [60.9–110.1] vs 60.3 [45.2–79.9], *p* = 0.01, respectively) (Fig. 1E, K). All other body-fluid biomarkers analyzed at baseline in this cohort including CSF concentrations of NfL, GFAP, T-tau, and sNfL, did not differ significantly between the groups (Fig. 1). Since sGFAP is age-dependent, and as patients with PIRA^+^ were significantly older, we performed ANCOVA with age as a covariate. After adjustment for age, sGFAP remained statistically significantly higher in CDW (*p* = 0.008) and in PIRA^+^ (*p* = 0.044).

**Fig. 2:**
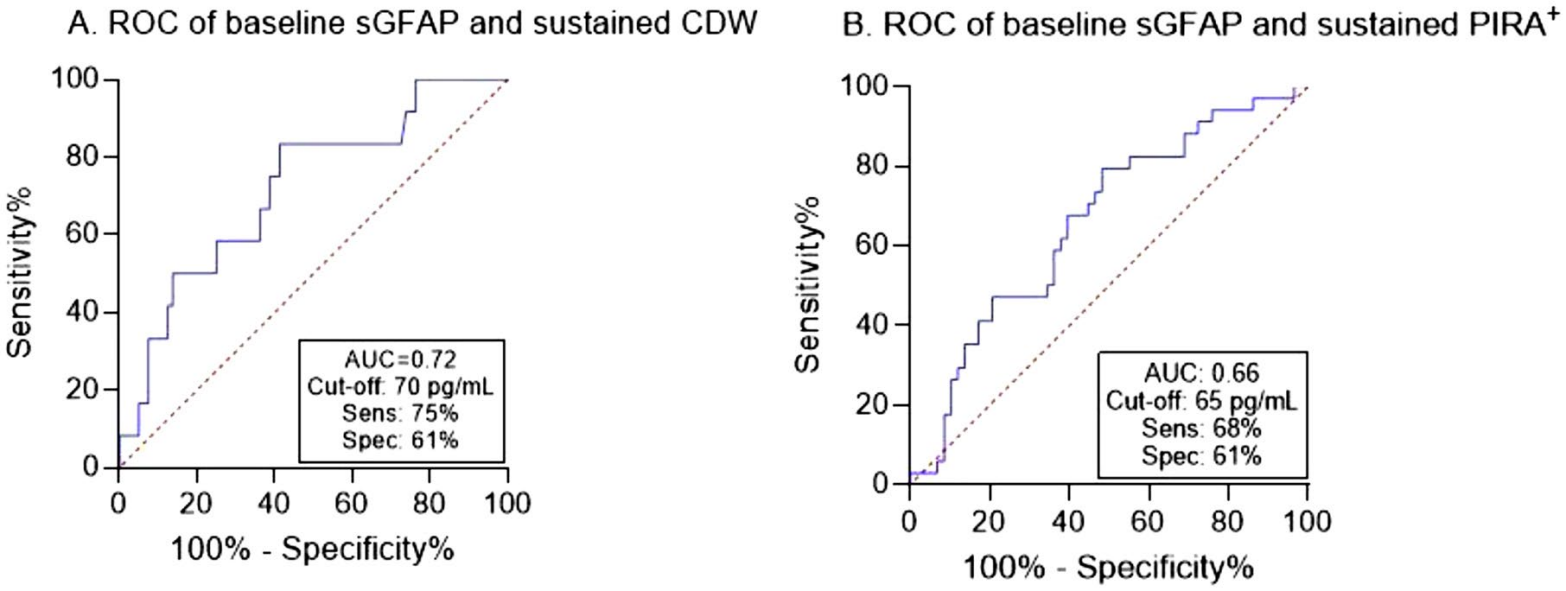
ROC curve showing performance of sGFAP levels at baseline in patients with early RRMS to discriminate between A. sustained CDW and non-CDW, and B. PIRA^+^ (determined by a composite score including sustained CDW, 9HPT, T25FWT, and SDMT) and non-PIRA^+^. ROC = receiver operating characteristic; sGFAP = serum glial fibrillary acidic protein; CDW = confirmed disability worsening; PIRA = progression independent of relapse activity; 9HPT = 9-hole peg test; T25FWT = timed 25 foot walk test; SDMT = single digit modalities test; AUC = area under the curve; Sens = sensitivity; Spec = specificity

Hereafter, we opted to focus on the predictive ability of sGFAP in terms of CDW and PIRA^+^. In a ROC-curve analysis in which the cohort was dichotomized by sustained CDW status, sGFAP had AUC of 72% (Fig. 2A). A sGFAP cut-off value of 70 pg/mL discriminated between patients who had sustained CDW and those who remained stable. In a univariable logistic regression analysis, sGFAP > 70 pg/mL was associated with higher odds of sustained CDW (OR 4.5, 95% CI 1.14–18.14, *p* = 0.032). In a multivariable logistic regression adjusted for age, sex, disease duration, roving EDSS, treatment strategy, and MRI measures (T2 lesions and CEL), sGFAP > 70 pg/mL retained its association with sustained CDW (aOR 16.6, 95% CI 1.72–160.3, *p* = 0.015). When determining PIRA as a composite score, sGFAP had an AUC of 66% to detect PIRA^+^, and the cut-off value of 65 pg/mL was found to be the most discriminatory cut-off value (Fig. 2B). Applying this cut-off value resulted in a sensitivity of 68% and specificity of 61% to detect patients at higher risk of PIRA^+^. In a univariable logistic regression, sGFAP > 65 pg/mL was associated with higher odds of developing PIRA^+^ during the study follow-up period (OR 3.2, 95% CI 1.3–7.8, *p* = 0.011) (Table 2). This result was retained in the multivariable analysis (aOR 4.3, 95% CI 1.44–12.86, *p* = 0.009).

**Table 2:** Univariable and multivariable logistic regression models for baseline sGFAP as well as covariates and prediction of PIRA^+^

	OR	95%CI	*p*-value
Univariable			
Age (y)	1.067	1.019–1.117	**0.006**
Sex (F)	1.85	0.69–4.92	0.22
Disease duration (y)	1.04	0.95–1.13	0.39
Roving EDSS	1.34	0.93–1.94	0.12
BL T2 lesions (≥ 10)	1.9	0.86–4.24	0.11
BL CEL (yes)	0.85	0.39–1.87	0.69
Exposure to heDMT (yes)	1.3	0.54–3.07	0.57
sGFAP > 65 pg/mL	3.2	1.3–7.8	**0.011**
Multivariable			
Age (y)	1.074	1.013–1.14	**0.016**
Sex (F)	1.32	0.4–4.32	0.65
Disease duration (y)	0.93	0.83–1.05	0.23
Roving EDSS	1.5	0.94–2.4	0.09
BL T2W lesions (≥ 10)	2.12	0.75–5.99	0.15
BL CEL (yes)	0.44	0.15–1.33	0.15
Exposure to he-DMT (yes)	1.5	0.45–4.9	0.51
sGFAP > 65 pg/mL	4.3	1.44–12.86	**0.009**

sGFAP = serum glial fibrillary acidic protein; PIRA = progression independent of relapse activity; BL = baseline; CEL = contrast-enhancing lesions; heDMT = highly effective disease modifying therapy;

Bold text indicates *p* values < 0.05

### Longitudinal dynamics of serum biomarkers to discriminate PIRA^+^ from stable MS

**Fig. 3:**
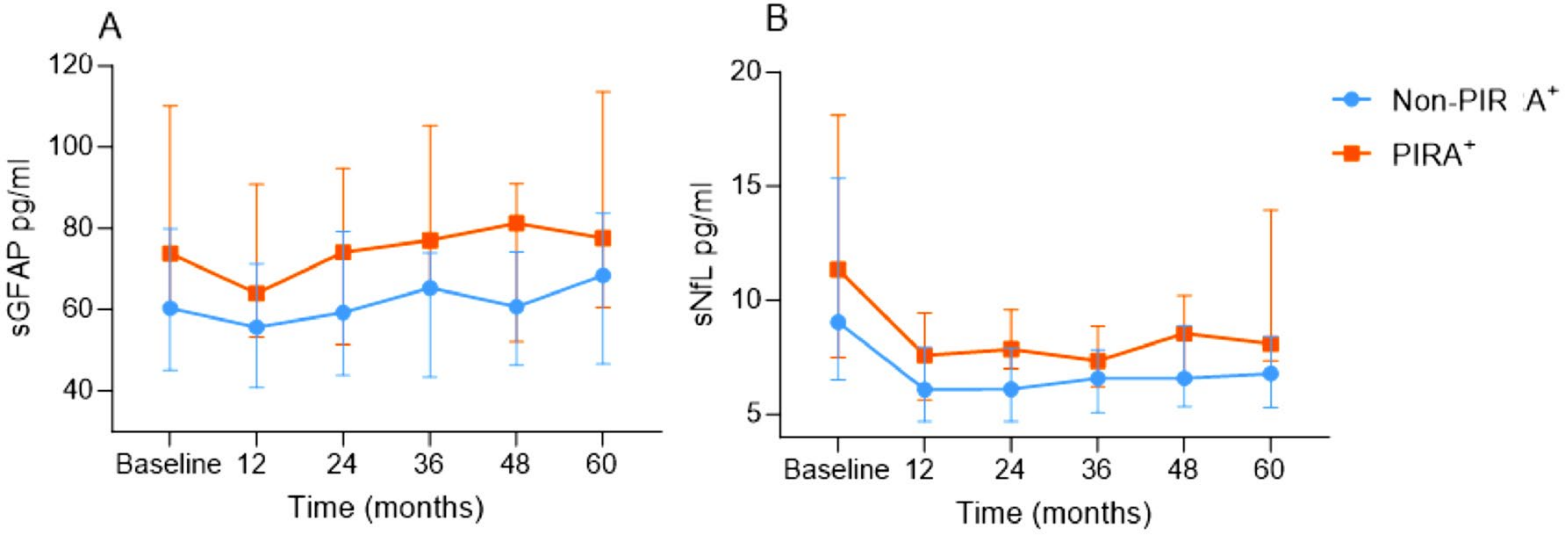
Longitudinal dynamics of A. serum GFAP and B. serum NfL measured at baseline, 12, 24, 36, 48, and 60 months in patients with PIRA^+^ vs. non-PIRA^+^. Symbols represent median, bars represent interquartile range. sGFAP = serum glial fibrillary acidic protein; sNfL = serum neurofilament light

Serum biomarkers were sampled longitudinally along the study follow-up (baseline [*n* = 92], 12 [*n* = 74], 24 [*n* = 57], 36 [*n* = 62], 48 [*n* = 73] and 60 months [*n* = 80]). Repeated measures of sGFAP levels showed that patients with PIRA^+^ during follow-up had higher sGFAP levels (20%, *p* < 0.001) along the whole follow-up compared to stable patients (Table 3; Fig. 3A) but over time, sGFAP concentrations were overall not significantly changed within groups (*p* = 0.18). After correction for multiple comparisons, levels of sGFAP at 36 months and 48 months significantly differed between PIRA^+^ and non-PIRA^+^ (adjusted *p* = 0.017 and adjusted *p* = 0.01, respectively). Repeated measures of sNfL levels demonstrated a significant reduction over time (*p* < 0.001), and patients with PIRA^+^ had higher levels compared to stable patients along the follow-up time (12%, *p* = 0.009) (Fig. 3B). After correction for multiple comparisons, sNfL concentrations significantly differed between PIRA^+^ and non-PIRA^+^ at 24 months (adjusted *p* = 0.017) and 60 months (adjusted *p* = 0.005). The longitudinal levels of sGFAP and sNfL in CDW and non-CDW were similar to the analysis with PIRA^+^ (supplemental Figure 2).

**Table 3:** Results of multivariable mixed linear models and the association of repeated log2-transformed serum biomarker measurements with PIRA^+^

	Estimate (95% CI)^a^	*p*-value
Model 1: sGFAP repeated measures as dependent variable		
Age (y)	1.01 (1.006–1.015)	** < 0.001**
Sex (F)	1.086 (0.99–1.19)	0.08
Roving EDSS	0.96 (0.92–0.99)	**0.025**
Disease duration (y)	1.22 (1.01–1.03)	** < 0.001**
Exposure to heDMT (No)	1.26 (1.15–1.37)	** < 0.001**
PIRA^+^	1.2 (1.1–1.31)	** < 0.001**
Model 2: sNfL repeated measures as dependent variable		
Age (y)	1.02 (1.013–1.023)	** < 0.001**
Sex (F)	0.92 (0.84–1.012)	0.088
Roving EDSS	0.99 (0.96–1.036)	0.968
Disease duration (y)	1.016 (1.006–1.025)	**0.001**
Exposure to heDMT (No)	1.15 (1.05–1.25)	**0.002**
PIRA^+^	1.12 (1.03–1.22)	**0.009**

PIRA = progression independent of relapse activity; CI = confidence interval; sGFAP = serum glial fibrillary acidic protein; EDSS = expanded disability status scale; heDMT = highly effective disease modifying therapy

^**a**^ Estimates are back-transformed

Bold text indicates *p* values < 0.05

### Association of baseline imaging biomarkers with PIRA

In the present analysis, baseline T2 lesions and the presence of CEL did not statistically significantly differ between patients with and without PIRA^+^ (Table 1). Baseline MY and BPF, RNFL and GCIPL did not significantly differ between patients with and without PIRA^+^. When applying previously published cut-off values, neither RNFL ≤ 88 μm (OR 2.03, 95% CI 0.63–6.6, *p* = 0.24) nor GCIPL < 77 μm (OR 1.15, 95% CI 0.31–4.24, *p* = 0.83) were associated with PIRA^+^.

### Sensitivity analyses

Next, we sought to investigate whether findings concerning CDW and PIRA^+^ were still valid when taking MRI activity into account. In total, six out of 40 patients (15%) with PIRA^+^ also had signs of inflammatory disease activity on MRI within three months apart from the PIRA^+^ event, i.e. 34 patients had PIRMA^+^. Similar to the analysis with PIRA^+^ as an endpoint, sGFAP significantly differed between PIRMA^+^ and non-PIRMA^+^ (*p* = 0.01). Furthermore, CSF GFAP also significantly differed between PIRMA^+^ and non-PIRMA^+^ (*p* = 0.005). None of the other biomarkers were statistically different in PIRMA^+^ vs. non-PIRMA^+^.

A ROC curve analysis revealed an AUC of 66% for sGFAP, and a cut-off value of > 65 pg/mL remained as the most discriminatory (not shown). A ROC curve analysis with CSF GFAP as the dependent variable revealed an AUC of 67%. A cut-off value of CSF GFAP > 225 ng/L was calculated to be the most discriminatory cut-off value. In a multivariable logistic regression, sGFAP remained independently and strongly associated with PIRMA^+^ (aOR 8.35, 95% CI 2.23–31.31, *p* = 0.002). Further, in a multivariable logistic regression model, CSF GFAP > 225 ng/L emerged as an independent predictor of PIRMA^+^ (aOR 5.2, 95% CI 1.51–17.82, *p* = 0.009).

## Discussion

In this study, we present data on eleven different body-fluid and imaging biomarkers, and their association with PIRA^+^ in patients with early RRMS. All data were collected prospectively during a follow-up period of 5 years. Since the only biomarker in our analysis to significantly differ between PIRA^+^ and non-PIRA^+^ was sGFAP, we particularly focused our further investigations on this specific biomarker. The key findings in our study are that baseline levels of sGFAP are higher in patients who developed PIRA^+^ during follow-up, and that high sGFAP (> 65 pg/mL) was independently associated with a 4.3-fold increase in the odds for PIRA^+^. Patients with PIRA^+^ tended to have higher levels of sGFAP when sampled repeatedly, but sGFAP essentially neither increased nor decreased along a period of 60 months. None of the other investigated biomarkers demonstrated significant associations with PIRA^+^, although longitudinal sNfL dynamics were on average higher in PIRA^+^ compared to non-PIRA^+^. In this regard, our results are in line with a previous report on the role of sGFAP and sNfL to detect and predict the risk of disease worsening [8].

In the present analysis, baseline sGFAP was more precise in detecting sustained CDW (AUC 0.72) compared to PIRA^+^ (AUC 0.66). This might be due to the fact that the PIRA^+^ composite score captures a considerably larger number of patients compared to CDW based on the EDSS alone (38.5 vs 12.5%).

PIRA has been recognized as the most common form of disability accrual in MS, challenging the traditional division of MS disease course into relapsing–remitting and progressive subtypes. Diverging from relapse-associated worsening (RAW), PIRA is characterized by a heightened propensity for severe disability [32], higher rate of cortical atrophy [33], and a generally unfavourable prognosis, particularly when manifesting early in the course of MS.

Importantly, in the present study, we opted to use a composite score to define sustained PIRA, including, in addition to the EDSS, the 9-HPT, T25FWT, and SDMT [6]. This strategy is thought to increase the overall accuracy to detect PIRA in patients with early RRMS, and compensate for the interrater variability that might affect determinations based on the EDSS alone. Interestingly, when testing sGFAP against CDW, we found comparable results to the analysis with PIRA^+^ as an endpoint.

The goal of identifying biomarkers that associate with smouldering MS remains essential. This will be particularly important in light of the ongoing efforts to develop new drugs that attempt to halt the process of smouldering MS disease. Particularly, smouldering MS is known to be at least partly driven by microglial activation and astrocytosis (pathological astrocyte activation) [2]. GFAP has been previously proposed as a reliable biomarker for disease severity in MS [16, 18]. In addition, sGFAP seems to reflect astrocytic damage also in patients with primary progressive MS [34], thereby highlighting a potential role for sGFAP in capturing neurodegenerative processes in MS. Moreover, sGFAP has been demonstrated to associate with microdamage in the normally-appearing white matter, as determined by diffusion tensor imaging [35]. Thus, it is not surprising that sGFAP stands out in our analysis as a biomarker that may capture smouldering neurodegeneration. This finding is in line with two recent large studies [8, 36], but contrasts several other studies that could not demonstrate such an association [22, 23, 37, 38]. This incongruity may arise from variations in cohort size, pre-analytical factors, assay platform, and/or divergent methodologies employed for assessing disease endpoints. Serum levels of NfL are known to reflect ongoing inflammatory disease activity and the resulting axonal damage, but the ability of sNfL to capture or predict disability accrual regardless of inflammatory disease activity has not been convincing [21, 39].

In the present cohort, longitudinal sGFAP concentrations were associated with PIRA^+^, which is in line with the study by Meier et al. [8], although the results in this study pertain mostly to CDW. This is in contrast to a previously published study that did not find an association between longitudinal sGFAP levels and PIRA status [23]. This might be due to a smaller cohort in that study, a shorter follow-up time with less sample time points, as well as the fact that the cohort in this study was limited to patients treated solely with natalizumab, thus limiting its generalizability. Another study investigated longitudinal measurements of sGFAP and did not find an association with disability progression independent of inflammation over time [22]. However, this study cohort was composed solely of patients with secondary progressive MS patients, rendering a comparison difficult.

In contrast, longitudinal sNfL levels in our study decreased after baseline, and remained stable below a median 10 pg/mL, even though PIRA^+^ had consistently higher levels than non-PIRA^+^. This finding highlights the ability of sGFAP to capture disability accrual independent of the focal acute inflammation that is often associated with elevated NfL concentrations.

Similarly to a previously published study by our group [40], CSF GFAP levels in the present cohort did not significantly associate with PIRA. However, after adjusting our analysis for relevant MRI activity three months apart from the PIRA event (PIRMA), CSF GFAP was indeed independently associated with PIRMA. This finding is in line with a number of previous studies [12, 41, 42]. GFAP is the predominant intermediate filament protein within astrocytes. It is recognized as the principal structural element underlying astrogliosis and serves as the primary protein component within chronic MS lesions [43]. After the development of an in-house method to measure GFAP in CSF [30], we demonstrated for the first time an association between CSF GFAP and disease progression in MS [12]. Further, a very recent study demonstrated a strong association between CSF GFAP and nonrelapsing progressive MS biology [42]. In that study, CSF GFAP was found to correlate with slowly expanding lesions (SELs), higher EDSS scores and lower thalamic volume. Interestingly, CSF GFAP was found to be expressed by a greater proportion of astrocytes from chronic active MS lesions compared with inactive lesions or control white matter [42]. Taken together, these and our findings reinforce the notion that GFAP may be more specific to the smouldering compartmentalized inflammation that is associated with PIRA.

In the context of neurodegenerative disorders, such as Alzheimer's disease (AD), the measurement of blood GFAP has demonstrated heightened efficacy compared to CSF levels in capturing amyloid-β (Aβ) pathology, particularly in the initial stages of the AD spectrum [44]. This observation deviates somewhat from the conventional understanding, wherein blood-based biomarkers for neurological conditions are generally regarded as approximations of CSF levels. Plausible rationale for this phenomenon lies in the integral role of astrocytes within the neurovascular unit, responsible for maintaining the integrity of the blood–brain barrier frequently compromised in MS [45]. Astrocytic endfeet extensively envelop brain capillaries, exerting regulatory influence over micro-vessel dilation and constriction [46]. Consequently, the direct release of GFAP from reactive astrocytes into circulation through this intricate pathway may account for the observed disparities. This is particularly important, as sGFAP has the additional advantage of being measured in an easily accessible compartment, rendering it suitable for longitudinal determinations in clinical practice.

One limitation to this study is a number of missing data concerning longitudinal serum sampling. However, we addressed this limitation by performing a mixed-effects model, taking missing values into consideration. Furthermore, we did not have data on BMI, which is known to influence sNfL and is believed to affect sGFAP as well, and did not calculate z-scores for sGFAP. However, in contrast to sNfL z-scores, sGFAP z-scores have not yet gained the necessary evidence to be applied on a broader basis.

Nevertheless, we adjusted our multivariable models for age and sex. Furthermore, none of the baseline biomarkers in the present cohort was associated with neither age nor sex. Our study population consisted of patients who received both low/moderate and high efficacy (most notably natalizumab) therapies from disease onset, although there were relatively few patients treated with anti-CD20 monoclonal antibodies. This is in contrast to the study by Meier et al. [8] which included mostly patients on anti-CD20 therapies as heDMTs but not natalizumab. In that sense, our study complements their results and adds important knowledge on the lack of effect of natalizumab on astrogliosis and aberrant astrocyte activation.

In conclusion, we demonstrate the clinical utility of measuring sGFAP concentrations at baseline and longitudinally to capture disability accrual independent of acute focal inflammation in patients with RRMS. Serum GFAP was the only biomarker to capture disease progression and was superior to other body-fluid and imaging biomarkers. Our study supports the inclusion of sGFAP measurements in clinical practice, as part of routine diagnostic and follow-up procedures. In that sense, measuring sGFAP might aid in the early identification of MS patients at greater risk of developing PIRA, who may particularly benefit from treatment strategies specifically directed against smouldering MS, if and when such therapies will exist.

### Supplementary Information

Below is the link to the electronic supplementary material.

Supplementary file1 (PDF 392 KB)

Supplementary file2 (PDF 616 KB)

## Data Availability

Anonymized data, not published in the article, will be shared on reasonable request from a qualified investigator.
