# Supplementary figures and images for: Association of serum glial fibrillary acidic protein with progression independent of relapse activity in multiple sclerosis
Source: J Neurol. 2024 Apr 26;271(7):4412–22. doi: 10.1007/s00415-024-12389-y (PMC11233378; doi:10.1007/s00415-024-12389-y)

**Supplemental Figure 1.** Correlations of baseline cerebrospinal fluid and serum biomarkers.

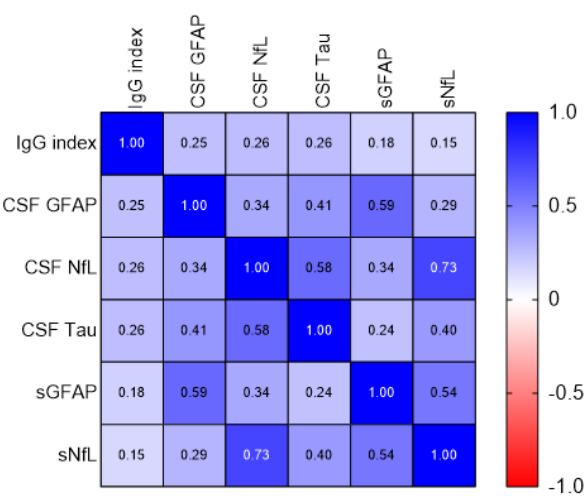

Supplement: Supplementary file 1 — Supplementary file1 (PDF 392 KB) [file 415_2024_12389_MOESM1_ESM.pdf]
