# Supplementary material for: Association of serum glial fibrillary acidic protein with progression independent of relapse activity in multiple sclerosis
Source: J Neurol. 2024 Apr 26;271(7):4412–22. doi: 10.1007/s00415-024-12389-y (PMC11233378; doi:10.1007/s00415-024-12389-y)
Supplement: Supplementary file 2 — Supplementary file2 (PDF 616 KB) [file 415_2024_12389_MOESM2_ESM.pdf]

Supplemental figure 2. Longitudinal dynamics of sGFAP and NfL in patients with CDW compared to patients who remained stable along the study follow-up

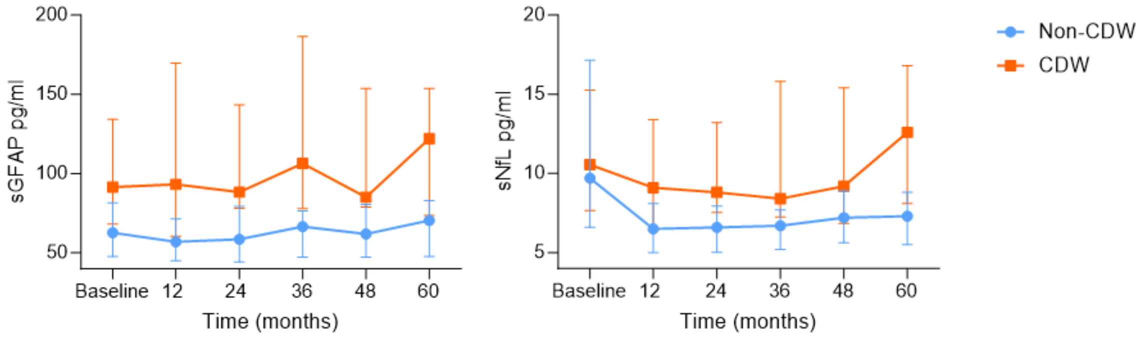

Supplemental Fig. 2 Longitudinal dynamics of A. serum GFAP and B. serum NfL measured at baseline, 12, 24, 36, 48, and 60 months in patients with CDW vs. non-CDW. Symbols represent median, bars represent interquartile range.
